# Craniotomy

**Published:** 1886-02

**Authors:** 


### CRANIOTOMY.

The Catholic Church has promulgated a decision that the destruction of a fœtus in parturition or otherwise, even when absolutely necessary to the salvation of the life of the mother, is never justifiable. That is, the physician must allow both mother and infant to die, when one might be saved

The Jewish Church, according to *The American Israelite*, permits the destruction of the child when it cannot possibly be born without sacrificing the life of the mother.

We really do not see what either one has to do with the matter. It is the province of the physician to judge of this. Metaphysicians should not attempt to meddle with physics. Men who have devoted their lives to abstract speculations are scarcely the ones to be entrusted with the decision of questions that involve life and death.

